# Scale-based screening and assessment of age-related frailty

**DOI:** 10.3389/fpubh.2024.1424613

**Published:** 2024-12-18

**Authors:** Xiao-Ming Wang, Yuan-Hui Zhang, Chen-Chen Meng, Lu Fan, Lei Wei, Yan-Yang Li, Xue-Zheng Liu, Shi-Chao Lv

**Affiliations:** ^1^First Teaching Hospital of Tianjin University of Traditional Chinese Medicine, National Clinical Research Center for Chinese Medicine Acupuncture and Moxibustion, Tianjin, China; ^2^Affiliated Hospital of Nanjing University of Chinese Medicine, Nanjing, Jiangsu, China; ^3^Department of Integrated Chinese and Western Medicine, Tianjin Medical University Cancer Institute and Hospital, Tianjin, China

**Keywords:** older people, frailty, screening, assessment, scale

## Abstract

As the population ages, the prevalence of age-related frailty increases sharply, which increases the risk of poor health status of older adults, such as disability, falls, hospitalization, and death. Across the globe, frailty is moving toward the forefront of health and medical research. Currently, frailty is believed to be preventable and reversible, so the early identification of frailty is critical. However, there are neither precise biomarkers of frailty nor definitive laboratory tests and corresponding clinical testing techniques and equipment in clinical practice. As a result, the clinical identification of frailty is mainly achieved through the widely used frailty scale, which is an objective, simple, time-saving, effective, economical, and feasible measurement tool. In this narrative review, we summarized and analyzed the various existing frailty scales from different perspectives of screening and evaluation, aiming to provide a reference for clinical researchers and practitioners to judge and manage frail older people accurately.

## 1 Introduction

As the life expectancy of the global population gradually increases with the advancement of medical treatment and the improvement of living standards, the problem of population aging is becoming increasingly serious, and how to face population aging positively has become the most important medical and social issue in the world (1). One of the major challenges facing an aging population is the increasing prevalence of age-related frailty, which is a state of reduced ability to cope with stimuli due to age-related declines in the physiological reserve capacity and function of multiple systems and organs (2). Several prospective cohort studies have shown that frailty is strongly associated with poor health and that frail older people are more likely to experience death (3), disability (4–6), falls (7), and hospitalization (2) than non-frail older adults. Although frailty poses a significant risk of adverse health outcomes in older adults, it is a dynamic and reversible disease, which means that it is preventable and controllable and that early recognition and interventions of frailty can halt its progression (2, 8). Early identification and diagnosis of frailty will help to maximize the reversal of its further progression, alleviate or delay underlying symptoms, control adverse clinical health outcomes such as recurrent hospitalization and death, maintain their functional status, and enhance their quality of life. Several studies have shown that timely recognition and intervention of frailty in the clinical setting or daily life can contribute to benefits for older adults (9, 10), and may even delay the onset of death in 3 to 5% of older adults (11). Clinical practice guidelines developed by the International Conference of Frailty and Sarcopenia Research (ICFSR) also recommend that adults 65 years old and older should be screened for frailty using a simple, validated, and rapid screening tool appropriate for the specific scenario and that all older adults considered to be frail or pre-frail should be further assessed for frailty (12). However, there are no standardized criteria regarding the selection of screening and assessment tools for frailty. Therefore, the development of efficient and practical screening and assessment tools for frailty should be a top priority in the field of frailty research. This paper provides an overview of the current state of research on screening and assessment tools for age-related frailty and the characteristics of commonly used frailty scales, with the aim of helping clinical researchers and practitioners to accurately judge and fine-tune the management of frail older adults.

## 2 Methodology

The search database was PubMed. The retrieval time node ranged from January 2000 to December 2023. The retrieval strategy was optimized with the use of Boolean logical operators. The retrieval formula is ((“Frailty/diagnosis”[Mesh] OR “Frailty/epidemiology”[Mesh]) OR ((Frailties[Title/Abstract]) OR (Frailness[Title/Abstract])OR (Frailty Syndrome[Title/Abstract]) OR (Debility[Title/Abstract]) OR (Debilities[Title/Abstract]))) AND ((“2000/01/01”[Date - Publication]: “2023/12/31”[Date - Publication])). Then, we imported the transcript of all retrieved literature into Endnote Application. After briefly reading the title and abstract, articles not related to the screening or assessment of frailty were excluded. The specific inclusion criteria were defined as follows: (1) papers covered a population of older adults aged ≥60 years; (2) the paper’s main topic was screening and assessment of frailty; (3) the full text was accessible; (4) paper was presented in English. The exclusion criterion was that the paper was on the pathogenesis or interventions for frailty or its association with other diseases. The process of screening the literature was done independently by two researchers, followed by cross-checking. In case of disagreement between the two researchers, a third person was consulted to assist in the judgment. Next, we read the remaining literature and used an Excel sheet to record the screening or assessment scales addressed in each paper, selecting those that appeared ≥150 times. Finally, we read the literature pertaining to the above scales carefully and used another Excel sheet to document the content, focus, measurement patterns, and application scenarios of each scale for subsequent categorization and summarization.

## 3 Current status of research on screening and assessment tools for frailty

At present, studies on screening and assessment tools for frailty have mostly focused on two areas: frailty-related biological markers and frailty-related scales. The development of frailty involves multiple complex pathophysiological processes such as chronic inflammatory responses, imbalances in energy metabolism, nutritional deficiencies, immune disorders, oxidative stress, and so on (13). In older frail patients, the levels of biological factors involved in these pathophysiologic processes are altered accordingly and can be biomarkers of frailty. To date, biomarkers of age-related frailty can be categorized as inflammatory response-related biomarkers (C-reactive protein, interleukin-6, tumor necrosis factor) (14–17), metabolism-related biomarkers (muscle growth inhibitor, 25-hydroxyvitamin D, insulin-like growth factor 1) (18–21), immune-related biomarkers (neutrophils/lymphocytes ratio, platelet/lymphocyte ratio and systemic immune-inflammatory index) (22), Oxidative stress-related biomarkers (8-dihydro-2′-eoxyguanosine, reactive oxygen species, and superoxide dismutase) (23, 24), and nutrient-related biomarkers (docosahexaenoic acid and vitamin B12) (25, 26). However, biomarkers are susceptible to a variety of factors, making these indicators potentially less stable. For example, the circulating level of insulin-like growth factor 1 can be affected by nutritional levels and genetic factors, and the ratio of neutrophil/lymphocyte is susceptible to factors such as acute illnesses and infections. In addition, some frailty biomarkers have gender specificity, such as C-reactive protein, interleukin-6, and muscle growth inhibitors, which makes it necessary to take gender into account when selecting biomarkers. Therefore, although the changes in biomarkers precede the appearance of the organism’s phenotype, and the objectivity and sensitivity of biomarkers are superior, their specificity, precision, stability, and reliability are weaker than those of frailty-related scales (27). In fact, due to the lack of definitive laboratory tests and appropriate clinical testing techniques and equipment (28), the frailty-related scales have become the most commonly used clinical tool for the identification and assessment of frailty (29).

The frailty scales are mostly based on the three conceptual models of frailty and are established by incorporating the clinical symptoms, signs and subjective feelings of the patient, which have the advantages of simplicity, time-saving, validity, economy and feasibility, such as: Fried Frailty Phenotype (FFP), Frailty Index (FI), Groningen Frailty Indicator (GFI), and so on. Three of the conceptual models described above are the Biological Phenotype, the Cumulative Health Deficit Model, and the Frailty Integral Model. In 2001, Fried et al. (30) proposed the concept of the Fried Frailty Phenotype (FFP) based on the theory of the Biological Phenotype, stating that frailty is a syndrome that meets three or more of the five phenotypic criteria, which is mostly centered on physical deterioration, together with a decrease in physical performance and muscular strength. The Cumulative Health Deficit Model suggests that the more health deficits are accumulated, the more severe the degree of frailty. Based on the Cumulative Health Deficit Model, Rockwood et al. (31) proposed the concept of the Frailty Index (FI) in 2005, which considered frailty as a complex unity of physiological, psychological, and social functioning, and a risk condition that develops as a result of the accumulation of multiple disorders due to multiple factors. The above two models are commonly used in existing studies and have been generally confirmed. Based on these two models, Gobbens et al. (32) proposed the Integral Model of Frailty (IMF), which further defines the operational definition of frailty as a dynamic and continuous process that includes somatic, psychological, and social aspects. Recently, WHO has proposed a new concept of “intrinsic capacity” based on healthy aging, which emphasizes the physiological and psychological dimensions of the individual, and is a longitudinal assessment that follows a trajectory rather than the traditional assessment of frailty at a cross-section or cut-off point. In a sense, intrinsic capacity evolves from frailty, and frailty is one of the components of the decline trajectory of intrinsic capacity (33). Another hybrid concept analysis of frailty described frailty as a dynamic and fluctuating inability to manage biopsychosocial and environmental stimuli that involves a decline in functioning and life changes, leading to a loss of autonomy and motivation, or poor health outcomes (34). Based on the above concepts, the frailty scales should address multiple dimensions such as somatic, psychological as well as social conditions. Frailty scales can be categorized as screening scales and assessment scales, and the two are often conflated in clinical practice. In fact, screening tools are not identical to assessment tools, and the emphasis of the two is not the same. The frailty screening scales focus on their operationalization, efficiency, and high sensitivity in order to screen older patients at risk of frailty or in a stage of frailty in a very short period of time, while the frailty assessment scales is more complex, focusing on high precision and support by reasonable biological indicators in order to determine more precisely the stage of frailty in which they are placed, and then to develop different treatment plannings according to their stages and risks (35).

Besides, the different screening and assessment tools count the different prevalence rates of frailty (36). A Meta-analysis showed that the prevalence of frailty within the same group was 12% using the FFP versus 24% using the FI (37). Another cross-sectional study among older Brazilians showed that the prevalence of frailty was 0.3% when assessed only in the physical domain of the Tilburg Frailty Indicator (TFI), 2.9% when assessed in both the physical and social domains, and 52% when assessed in a combination of all three domains: physical, social and psychological (38). Although a variety of geriatric frailty scales have been developed, there is still no recognized gold scale for assessing geriatric frailty, and translation from research to clinical practice remains a challenge in the future (39).Next, this article summarizes and analyzes the existing commonly used frailty scales in terms of their screening and assessment roles, and classifies each scale according to its content, focus, measurement mode, and application scenarios, so that clinicians or researchers can use the most appropriate frailty scales according to their characteristics, thus achieving the goal of early screening, early assessment, and early intervention of frailty, and reducing a series of adverse outcomes.

## 4 Screening scales for age-related frailty

The purpose of frailty screening is primarily to make a quick diagnosis, which is performed in all groups of older people, to identify those who are at high risk of frailty or already in a state of frailty through the use of simple tests. Here, we summarize and generalize the advantages and disadvantages of some commonly used screening scales for age-related frailty.

### 4.1 Frail scale (FS)

The FS is a clinically applicable self-screening scale for frail older adults proposed by the experts of the International Academy on Nutrition and Aging (IANA) (40). FS is a simple patient self-reported questionnaire containing only 5 items as follows: fatigue, increased sense of resistance, decreased activity, multimorbidity co-morbidity, and weight loss. It quickly categorizes the state of an older person into 3 types, among which those who meet 3 or more items are frail, those who meet 1 to 2 items are pre-frail, and those who do not have any of the 1 items are in a healthy state (41). FS can be easily mastered by healthcare professionals and has a high degree of maneuverability and screening efficacy, which has been translated into many languages and widely used worldwide (42–44). In addition, FS has high specificity and sensitivity. A survey of the Chinese version of the adaptation of FS among 1,235 Chinese community-dwelling older adults showed that the sensitivity of FS was as high as 86.96% while the specificity was as high as 85.64% (42). Another study, which conducted on 308 Chinese older patients aged 60 years and above, showed that FS had a sensitivity of 85.9% and a specificity of 72.5%, and noted that FS was convenient and time-saving, which could be used for the initial screening of frail patients to improve work efficiency (45). A cross-sectional study conducted by Aprahamian et al. (46) in a geriatric outpatient clinic showed that the sensitivity of FS was 54% and the specificity was 73% and suggested that FS could be selected as a screening tool for frailty because of its significant time and cost benefits. FS can be used not only to screen for frailty but also to predict adverse outcomes in older adults. FS is a valid predictor of mortality in older adults over 10 years, according to a cohort study among older adults aged 65 years and older (47). In a longitudinal study of women’s health in middle age in Australia, FS predicted the incidence of disability in women from middle age to old age over the next 15 years (48).

FS is entirely self-reported, without any objective measures, and can even be completed by telephone without face-to-face inspection, which makes it simple and easy to administer (49). The simplicity and ease of FS increases the convenience and completion rate of frailty screening, reduces the cost of screening, helps to carry out the development of frailty review, and is worthy of clinical application. However, FS suffers from a certain amount of information bias due to its complete reliance on patient self-reported outcomes, and special attention should be paid to this point in clinical applications.

### 4.2 Clinical Frailty scale (CFS)

The CFS is a frailty screening tool developed in 2005 by Rockwood et al. (31) for use in the Canadian Health and Aging Study. The original CFS contained 4 dimensions: physical activity, mobility, physical function, and energy status, which categorized older adults’ health status into 7 levels. With further research on geriatric frailty, the scope of CFS was expanded to co-morbidities, functional status, and cognitive ability domains, increasing the classification to 9 (50). The specific levels of CFS are as follows: Very Fit, Fit, Managing Well, Living with Very Mild Frailty, Living with Mild Frailty, Living with Moderate Frailty, Living with Severe Frailty, Living with Very Severe Frailty, and Terminally Ill, wherein levels 5 and above are defined exactly as a frailty state. A follow-up study of 210 acutely hospitalized older patients with adverse health outcomes found that both CFS and FS could identify older adults at risk for hospitalized adverse health outcomes and could be used as an easy screening tool for frailty; however, CFS demonstrated higher sensitivity than FS (89.6% vs. 54.6%) (51). Another cross-sectional study conducted in China also confirmed that the sensitivity of CFS was superior to FS as a screening tool for age-related frailty, both in all patients (94.1% vs. 63.0%) and in patients from different wards (91.8–98.5% vs. 58.0–65.7%) (52). In their study of the association between CFS and in-hospital mortality in patients with Corona Virus Disease 2019 (COVID-19), Sablerolles et al. (53) found that the in-hospital mortality was significantly higher in frailty patients (CFS 6–9) than in healthy patients (CFS 1–3) and that the grade of CFS was negatively correlated with the health status of the patient. A meta-analysis indicated that CFS could predict in-hospital mortality in acutely ill older patients and is a reliable predictor of short-term mortality in older patients presenting to the emergency department (54).

CFS combines clinical judgment with objective measures and can be used not only to predict the need for institutional care or the incidence of death, but also to assess specific domains including co-morbidities, functioning, and cognition, making it a widely used screening tool for frailty (55). Because of its simplicity, rapidity, and accurate ability to predict adverse outcomes, CFS is often considered the most desirable tool for geriatric frailty screening in emergency medicine (56). In addition, CFS is highly sensitive to symptoms associated with frailty syndrome, which makes it also useful for assessing and stratifying the management of frailty (57). However, the completion of CFS needs to be based on clinical diagnosis combined with the interpretation of clinical parameters, which requires that the user should be a medical staff with a certain medical knowledge base, which limits the popularization and application of CFS to a certain extent.

### 4.3 Edmonton Frailty scale (EFS)

The EFS is a multidimensional screening scale for frailty developed by ROLFSON et al. (58) for non-specialists without specialized training in geriatrics based on the traditional frailty phenotype. The EFS consists of 9 dimensions and 11 entries as follows: (1) Cognitive function (unable to complete the clock drawing test successfully); (2) Functional performance (needing help from others in daily activities); (3) General health (self-assessment of health and the number of hospitalizations in the last year); (4) Independence (unable to complete manual labor alone, unable to walk 2 flights of stairs or walk 1,000 m); (5) Social support (unable to seek outside support successfully when encountering problems); (6) Medication status (being on 5 or more prescription medications at the same time, forgetting to take medication); (7) Mental status (depression); (8) Nutritional aspects (unintentional and significant weight loss recently); (9) Self-control (urinary and fecal incontinence). The EFS has a maximum score of 17, with a score of 0 to 4 indicating no frailty, 5 to 6 indicating sensitive individuals prone to frailty, 7 to 8 indicating mild frailty, 9 to 10 indicating moderate frailty, and 11 or more indicating severe frailty. EFS covers all domains of frailty and is highly correlated with other frailty scales (59). A study of the differences in the prevalence of frailty calculated by five frailty screening tools showed that the prevalence of frailty screened by the EFS was 25.2%, which was most similar to the prevalence of frailty of 27.6% after integrating the five frailty screening tools mentioned above, suggesting that the accuracy of EFS screening was high (52). In addition, the above study found that the EFS had the highest specificity for the assessment of frailty in surgical wards at 98.1%. EFS is commonly used in the identification of geriatric frailty prior to surgery and helps to stratify the risks and identify potentially modifying factors, which makes it have a higher feasibility rating in the surgical setting (60). A prospective study in people aged 70 years and older undergoing major abdominal surgery showed that the EFS has good reliability and validity and can be used as a preoperative assessment tool to predict the risk of surgical complications in older adults (61). In a study conducted by McIsaac et al. (62), it was found that although the accuracy of assessments of postoperative risks using the modified Fried Index (mFI) and the EFS was similar, the EFS had the advantage of a shorter time-consuming and greater patient acceptance, and should be recommended for clinical use.

EFS can be completed within 5 min, with high acceptance by both investigators and respondents. It is easy to operate and can be used by professionals or even non-professionals in multiple departments. It has a wide range of applications venues, which can be used in medical settings such as emergency, outpatient, and hospital wards, as well as in non-medical settings such as the community and the home, making it a reliable screening tool for geriatric frailty (63). However, EFS uses only one question to assess the specifics of the social support domain, disputing the comprehensiveness of the social frailty screen.

### 4.4 Fried Frailty phenotype (FFP)

FFP is a phenotype derived by Fried et al. (30) from observing and tracking the follow-up to validate adverse outcomes in 5317 older adults aged 65 years or older who participated in the U.S. Longitudinal Cardiovascular Health Study, which explains why FFP was also called the Cardiovascular Health Study Index (CHS). The entries of FFP consist of 5 self-reported symptoms combined with biologically measured signs. The details of its entries are as follows: significant loss of body mass (unintentional weight loss of more than 4.5 kg or more in the past 1 year), weakness (low grip strength in both hands), fatigue (self-reported to be more easily fatigued in the last 6 months), slowness of the body (significant slowing of the walking speed), and physical inactivity (sedentary and physically inactive). Among them, those who fulfill 3 or more are defined as frail, those who have 1 or 2 are defined as pre-frail, and those who do not have any of the above 5 are defined as non-frail. FFP can not only measure the physical frailty status of older adults but also reflect the mental health status of the older adults and obtain more objective and accurate data, which is the most popular and widely used frailty measurement tool in the clinic (12). Results of a survey conducted among cancer patients showed that FFP had a sensitivity of 92% and a specificity of 41% for screening for frailty (64). FFP is widely applicable and its reliability and validity have been validated many times (65). FFP has now been shown to have predictive value for adverse health outcomes in a diverse range of older adults, including hospitalized and general older adults (66).

FFP is excellent for initial stratification for risks of the older population based on different characteristics (i.e., robust, pre-frail, and frail), without the need for an initial clinical assessment, and can be applied at the first patient contact (67). However, FFP focuses on the physiological level of assessment, lacks social, psychological, environmental, and multiple disease factors, and the implementation of some items (e.g., grip strength, step speed, etc.) requires trained personnel and specialized tools (68). The characteristics of FFP described above make it inappropriate for older adults with cognitive impairment, psychiatric disorders, impaired functioning, or in the acute phase of illness, which also make its applicability limited to hospitals, communities, and nursing facilities. In addition, the 5 phenotypic criteria of frailty allow for different ways of measuring them, and many previous studies have adapted their measurements, which have been confirmed to lead to differences in measurement effects (69). Therefore, future studies should report all the details about how the phenotypic criteria of frailty are measured in order to facilitate the interpretation of the results.

### 4.5 Other common screening scales for frailty

In recent years, more and more frailty screening tools have been developed as a result of the progress of frailty research. For example, in 2007 Ensrud et al. (70) found that data collected using the Study of Osteoporotic Fractures (SOF) Index was independently associated with FFP-predicted frailty-related adverse health outcomes, and subsequently, the SOF index became one of the screening tools for frailty; the Kihon Checklist (KCL) was proposed by the Japanese government for the implementation of the long-term care insurance system (71); and the Simple Self-Assessment Screening Tool—the Vulnerable Elders Survey-13 (VES-13) was created by Saliba et al. (72). The characteristics of common frailty screening scales are summarized in Table 1 and compared from nine perspectives, including entries, time required, content, and so on (30, 31, 40, 58, 70–76).

**Table 1:** Overview and comparison of screening scales for age-related frailty.

Scale	Year	Country	Items	Time	Contents	Diagnostic criteria	Characteristics	Measure methods	Application site
FS (40)	2008	America	5	15–30s	Fatigue, resistance, ambulation, illness, Loss of weight	Satisfying: ≥3 items	Simple and time-saving. One-dimensional, only focusing on the physical aspect. Subjective.	Self-screening	Hospital, community
CFS (31)	2005	Canada	9	<5 min	The illustrated entries assessing the physical activity, mobility, physical functioning, energy status, co-morbidities, and cognition	Satisfying: ≥ levels 5	Fast. Accurate prediction of adverse outcomes. Professional medical knowledge is required.	Doctor’s clinical judgment	Hospital, community
EFS (58)	2006	Canada	9	<5 min	Cognition, basic health status, independence, social support, drug use, nutrition, emotion, function, incontinence	Satisfying: ≥ 7 scores	Simple and fast. Strong predictive validity of surgical risk. Easy to be accepted. Poor comprehensiveness because it only has one indicator assessing areas of social support.	Clinician, non-professionals’ judgment	Hospital
FFP (30)	2001	America	5	<10 min	Weight loss, slow pace, decreased grip strength, low physical activity, fatigue	Satisfying: ≥ 3 items	Objective and accurate. Tedious and time-consuming. Professional measuring tools are required. One-dimensional, only focusing on the physical aspect.	Doctor’s clinical observation	Hospital, sanatorium, community
SOF Index (70)	2007	America	3	<5 min	Weight loss, exhaustion, and unable to rise from chair 5 times	Satisfying: ≥2 items	Simple to operate. Poor comprehensiveness, because it only includes the physical level. Poor specificity.	Self-screening, assessment	Community
KCL (71)	2007	Japan	25	<15 min	7 areas: physical function, nutrition, feeding, social activity, memory, mood, and lifestyle	Satisfying: >0.25	Strong specificity with a separate frailty critical value in each dimension. Accurate. Time-consuming.	Self-screening	Community
VES-13 (72)	2001	America	13	<5 min	4 areas: activities of daily living, physical function, self-rated health, and one question on age	Satisfying: ≥ 3 scores	Simple and time-saving. Strong predictive validity of disability and death. Widely used in older patients with tumors.	Self-screening	Community
MFST-HP (73)	2016	Netherland	15	-	3 areas: physical function, psychological items, and social items. (the higher the score, the more serious the degree of frailty)	Satisfying: ≥ 6 scores	Good reliability. Better performance for excluding non-frail states. Poor ability to predict adverse health outcomes.	Doctor’s clinical judgment	Hospital
PRISMA-7 (74)	2008	Canada	7	<10 min	3 areas: basic demographic characteristics, social support, and activities of daily living	Satisfying: ≥3 scores	High sensitivity. Poor specificity. Poor ability to predict adverse health outcomes.	Self-screening	Community, outpatient department, emergency department
GFST (75)	2012	France	6	<5 min	2 areas: doctors’ clinical judgment and self-reported decline in physical function, such as living alone, weight loss, fatigue, mobility difficulties, memory loss, and slow pace	Doctors identify frailty based on questionnaires	Time-saving. Helpful for general practitioners to make clinical decisions. Subjective because it depends on the clinical decision of the general practitioners.	Doctor’s clinical judgment	Community
SPPB (76)	1994	America	3	-	Walking speed test, repeated chair stands test, and balance test	Satisfying: ≤ 6 scores	Simple to operate. Poor specificity. Poor comprehensiveness, because it only includes the physical level.	Doctor’s clinical judgment, non-professionals’ judgment	Community, outpatient departmen

CFS: Clinical Frailty Scale; EFS: Edmonton Frailty Scale; FFP: Fried Frailty Phenotype; FS: FRAIL Scale; GFST: the Gérontopôle Frailty Screening Tool; KCL: Kihon Checklist; MFST-HP: the Maastricht Frailty Screening Tool; PRISMA-7: the Program of Research to Integrate the Services for the Maintenance of Autonomy-7; SOF Index: Study of Osteoporotic Fractures Index; SPPB: the Short Physical Performance Battery; VES-13: Vulnerable Elders Survey-13.

## 5 Assessment scales for age-related frailty

Rapid screening should be followed by further precise assessment of all older adults in pre-frail and frail states. An accurate assessment to evaluate which state of frailty a frail older adult is in can help to predict poor health outcomes better and facilitate the development of individualized treatment and management plans for frailty patients. Recently, there have been a large number of studies devoted to the development of objective quantitative frailty assessment tools (77). Since these assessment tools are not limited to questionnaires and there are differences in the consistency of the assessment tools, places of application, populations administered, and dimensions assessed, the results of the assessment of frailty cannot yet be judged uniformly. Therefore, we will next summarize the characteristics of the commonly used assessment scales for age-related frailty.

### 5.1 Frailty index (FI)

FI is a classic tool for assessing frailty in older adults developed by Mitnitski et al. (78) based on the cumulative deficit model. It covers multiple dimensions such as physical, psychology, cognition, and social functioning, and contains 30 to 70 evaluation items, the specific content of which is variable. Since there are no standardized criteria for the content of its items, researchers can choose their own entries according to their own research purposes. Although FI lacks specific variables that are uniformly standardized, the stability of FI is supported by the fact that FI consisting of different numbers and types of deficient items yields similar assessment results in different populations or research settings. Generally speaking, when FI ≥ 0.25, it implies frailty; when FI < 0.12, it implies non-frailty; when FI is between 0.12 and 0.25, it implies that the older adults are in the pre-frail stage (79). The sensitivity and specificity of FI in identifying frailty was 94.8 and 87.0% in all patients, 96.4 and 88.8% in patients on the cardiology ward, 95.9 and 81.1% in patients on the non-surgical ward and were 89.6 and 89.5% on the surgical ward (52). FI can be used not only for the screening of debilitation but also for the assessment of debilitation. FI is the first tool to successfully quantify the frailty state of older adults and has been widely used in several countries due to its good reliability and validity (80). FI is strongly associated with negative health-related outcomes (including mortality) and with deterioration in disease-specific health status, which makes it a good predictor of clinical prognosis (81, 82). It has been found that FI can be utilized to evaluate the role of musculoskeletal disorders on frailty, rather than just being a categorical variable (83). In addition, FI has important applications in reflecting health service needs, public health management, and interventions. During the period of the COVID-19 pandemic, the use of an electronic version of the FI to assess frailty helped clinicians make decisions by identifying patients most likely to require ICU (intensive care unit) admission and those with a poor prognosis (84).

By focusing on the cumulative number of individual health deficits and integrating multiple complex health information into a single indicator, FI breaks through the limitation of a single variable describing the functional status, and can better assess the overall health status of older adults. With its advantages of multidimensionality, continuity, and objectivity, FI is suitable for frailty assessment in almost all environments. However, the establishment of FI requires a large amount of clinical information, while obtaining a large amount of clinical information is laborious, extremely cumbersome, and time-consuming, which is a major challenge in the use of FI for the assessment of frailty.

### 5.2 Comprehensive geriatric assessment (CGA)

CGA was conceptualized for the development of a scientific rehabilitation training program for older adults in the 1940s by Marjory Warren (85). As the population ages, the application of CGA continues to extend and becomes a common method of assessing and treating older patients with frailty or loss of function (86). CGA focuses on comprehensive assessments of somatic function, cognitive function, psychology, and social/environmental factors in older adults, thereby identifying and quantifying the degree of frailty, and providing the basis for subsequent frailty intervention strategies and comprehensive care, which is conducive to the early reversal of frailty, the slowing down of the deterioration process of frailty, and the improvement of health outcomes (87). A systematic evaluation that included 22 studies involving 10,315 patients showed that patients in the group that took interventions based on CGA had a lower likelihood of death or worsening of their condition and a higher likelihood of cognitive improvement compared to the group that took conventional medical care (88). Lee et al. (87) found that a CGA-based intervention program for a frail population could potentially promote healthy aging in community-dwelling older adults, with sustained health benefits of up to 1 year for them. Mazya et al. (89) conducted a trial of dynamic geriatric assessment-frailty intervention, in which the control group of the study received conventional treatment and humanistic care while the intervention group received dynamic assessment by CGA and multidisciplinary team interventions (including medication adjustments, exercise, and dietary advice, etc.) in addition to conventional care. After the 24-month intervention, the proportion of patients in the intervention group who were pre-frail was significantly higher than in the control group, suggesting that more patients with chronic diseases or co-morbidities in the intervention group moved from frailty to pre-frailty or strong than that in the control group. With the help of CGA, a comprehensive and scientific assessment of frailty can be made and a personalized medical intervention plan for the older adults can be developed to slow down the process of frailty by healthcare professionals.

CGA can accurately judge the health status of older adults, assess the degree or stage of frailty, identify its causes or triggers, and provide suggestions for its preventive or therapeutic measures, which can help in making risk stratification and clinical decisions for frailty. However, as the CGA is a multidimensional and interdisciplinary diagnostic and therapeutic process that emphasizes a multidimensional and comprehensive risk factor exploration and assessment, it requires a large amount of manpower, energy, and time, which is inconvenient in practice and is only applicable to the hospital healthcare environment (90). Furthermore, despite the fact that many studies have shown a large advantage of CGA for the assessment of frailty, most of these studies were conducted on small samples of older adults within a single institution or region, resulting in poor accuracy of the results of these studies, which still need to be further validated (91).

### 5.3 Groningen Frailty Indicator (GFI)

GFI is a widely used frailty screening tool developed by Steverink et al. (92) in 2001. The GFI consists of the physical dimension (mobility, multiple health problems, physical fatigue, vision and hearing), the psychological dimension (depressed mood and anxiety), the cognitive dimension (cognitive dysfunction), and the social dimension (emotional isolation), with a total of 15 entries. The 15 entries of the GFI are all dichotomous questions, with each score set at 0 or 1. Higher scores on the GFI indicate more severe frailty, with those scoring ≥4 diagnosed as moderately or severe frailty (93). A study comparing the ability of four frailty screening tools to predict frailty-related adverse outcomes showed that the sensitivity of the GFI in predicting the frailsty adverse outcomes of death and hospitalization was 76.2 and 63.9%, respectively, and the specificity was 42.1 and 50.3%, which suggests that the GFI has a higher sensitivity and a poorer specificity (94). When the Chinese version of the GFI was used to screen for pre-frailty and frailty in 350 Chinese community-dwelling older adults, it demonstrated good internal consistency, with a Cronbach’s alpha coefficient of 0.87, a re-test reliability of 0.87, and the concurrent validity between the GFI and the Fried frailty phenotype of 0.76. suggesting that the GFI is a reliable and valid tool for pre-frailty and frailty screening in community-dwelling older adults (95). In addition, GFI can accurately predict total healthcare costs for the following year and can help healthcare professionals allocate healthcare resources (96).

GFI has been widely used in many countries such as Germany, Italy, France, and so on, and it is suitable for use in different assessment environments, such as communities, nursing homes, and healthcare facilities (97). However, compared with other scales, GFI focuses more on physical indicators such as physical strength, functioning, and health status, and does not adequately take into account psychological, cognitive, and social aspects, which may make the results of the GFI assessment slightly less integrated and comprehensive.

### 5.4 Other common assessment scales for frailty

As the prevalence of frailty increases, more and more frailty assessment tools are being developed to make individualized and precise interventions for frail patients. For example, the Comprehensive Frailty Assessment Instrument (CFAI), which was first included in environmental assessment, was developed (98) and the Rapid Geriatric Assessment (RGA), which improves on the cumbersome assessment process of the CGA, was also created (99). A comparison of various common frailty assessment scales is shown in Table 2 (78, 86, 92, 98–101).

**Table 2:** Overview and comparison of assessment scales for age-related frailty.

Scale	Year	Country	Dimension	Item	Time (min)	Contents	Assessment criteria	Characteristics	Measuremethod	Application site
FI (78)	2001	Canada	4	30–70	20–30	Physical functions, psychological aspects, cognitive ability, and social functions	Non-frailty: FI: < 0.12. Pre-frailty: FI: 0.12–0.25. Frailty: FI ≥ 0.25.	A broad scope focusing on overall health assessment. Strong predictive validity. Tedious and time-consuming.	Doctor’s clinical observation	Hospital, community
CGA (86)	1989	America	6	30+	<15	General medical assessment, physical function, psychological status, social behavior ability, environmental health, and other assessment such as: diet health	A continuous score. Frailty: scores >0.25. The higher the score, the more severe the frailty.	Focusing on the older inpatients. Widely recognized and applied. Complex content without an uniform standard.	Doctor’s clinical observation	Hospital
GFI (92)	2001	Netherland	4	15	<15	Physical functions, psychological aspects, cognitive ability, and social functions	Frailty: scores ≥4. The higher the score, the more severe the frailty.	Simple. Uncertain predictive ability for adverse health outcomes.	Self -assessment	Community
CFAI (98)	2013	Belgium	4	23	<15	Physical functions, psychological aspects (mood and emotion), social functions (social relations and social support), environment	Mild frailty: scores: 20–40. Moderate frailty: scores: 41–50. Severe frailty: scores: 51–97.	Simple to operate. Uncertain assessment of social functions.	Self -assessment	Community
RGA (99)	2015	America	4	18	<5	Degree of frailty, nutritional status, degree of anorexia and cognitive impairment	It includes 4 scales: the FS, the SARC-F, the SNAQ, and the RCS. Each scale is scored separately.	Time-saving. High degree of reliability. Several entries are difficult to understand and memorize.	Doctor’s clinical observation	Hospital
TFI (100)	2010	Netherland	3	15	<15	Physical functions, psychological aspects, and social functions	Frailty: scores ≥5. The higher the score, the more severe the frailty.	Simple. Comprehensive. Subjective.	Self -assessment	Hospital, sanatorium, community
FRAIL-NH (101)	2015	America	4	7	<10	Physical functions, nutritional condition, co-morbidities, and self-care ability	The higher the score, the more severe the frailty.	Simple. Time-saving. Good predictive validity for adverse health outcomes.	Doctor’s clinical observation	Hospital, sanatorium

CFAI: Comprehensive Frailty Assessment Instrument; CGA: Comprehensive Geriatric Assessment; FI: Frailty Index; FRAIL-NH: Fatigue, Resistance, Ambulation, Incontinence/Illness, Loss of weight, Nutritional approach, and help with dressing; FS: FRAIL Scale; GFI: Groningen Frailty Indicator; RCS: Rapid Cognitive Screen; RGA: Rapid Geriatric Assessment; SARC-F: the Simple Five item Scoring Scale for Sarcopenia; SNAQ: Simplified Nutritional Appetite Questionnaire; TFI: Tilbury Frailty Indicator.

## 6 Conclusion

Frailty is an emerging global health burden with significant implications for clinical practice and public health. With the rapid growth of an aging population, the prevalence of frailty increases year by year. Frailty is a dynamically changing clinical state, with the pre-frailty phase showing potential reversibility. The early recognition of frailty and appropriate interventions for it can help slow or even reverse the process of it and reduce the risk of adverse outcomes. In order to achieve “healthy aging” centered on wellness, we need to improve the rate of the early identification of frailty for its early precise intervention. Although there are approximately 67 screening and assessment tools for frailty internationally and and there is a trend toward an increase in the number of such tools (77), different screening and assessment tools have more significant differences in conceptual basis, clinical utility, program content, and place of application. As a result, there is still considerable debate as to what is the best scale for screening and assessing frailty.

**Figure 1:**
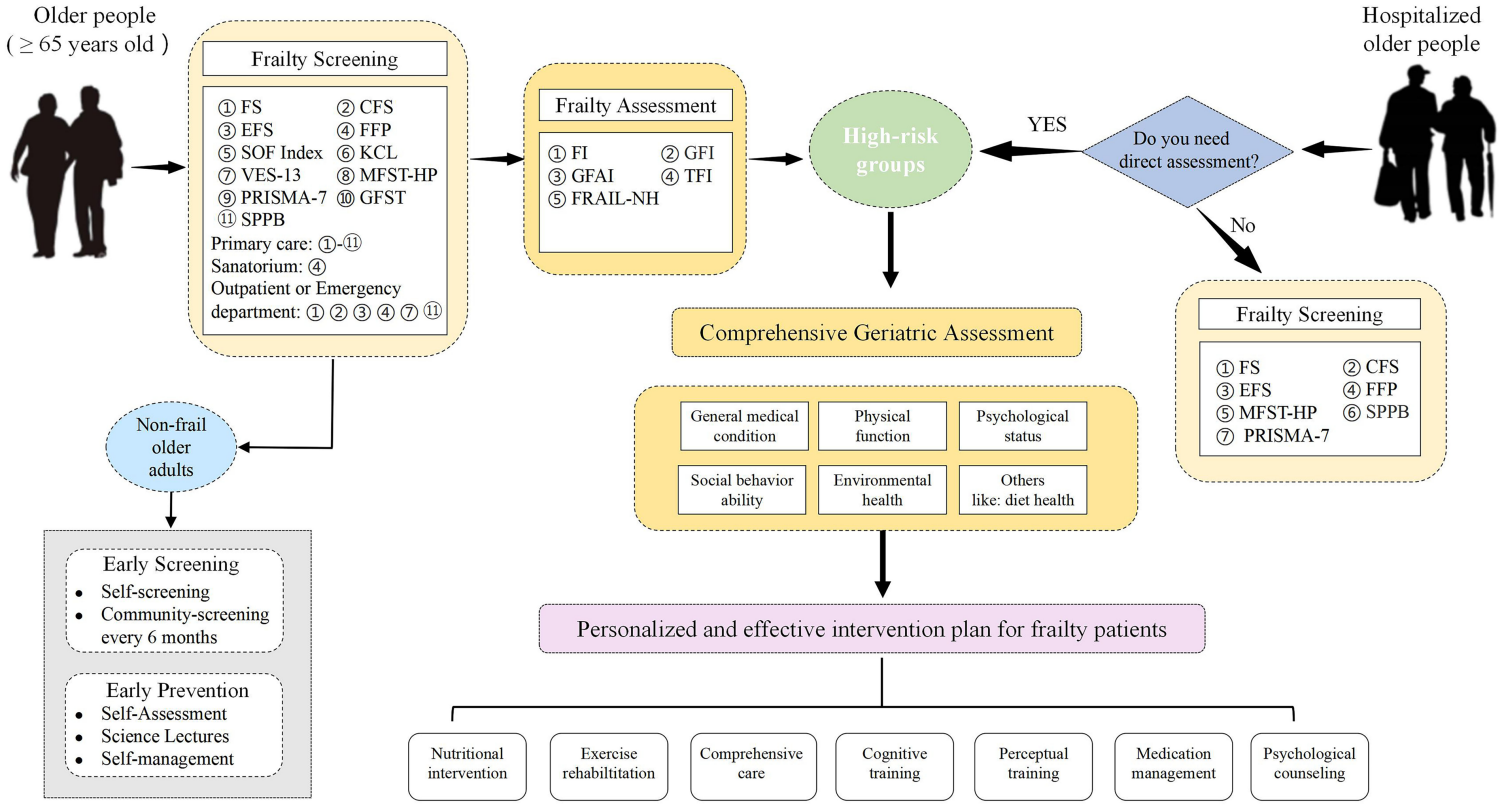
The process of screening and assessment for the frailty in different types of older people. The development of frailty can be slowed or even reversed by the early recognition, accurate assessment, and timely interventions for frailty, which will reduce the strain of frailty on health care systems around the world and promote healthy aging of the global population. In the future, we still need to explore objective, simple, time-saving, effective, economical, and feasible scales that can accurately identify and assess frailty in clinical practice.

Strictly speaking, frailty screening scales and assessment scales have different requirements and should not be confused. Screening scales need to be simple, quick, and highly sensitive to frailty, which allows clinicians to recognize frailty quickly. Assessment scales require high accuracy, utility, and support of sound biological theories, which allows clinicians to identify the stages of frailty in older adults accurately and predict the occurrence of adverse health events in frail older adults, such as falls, cognitive deficits, loss of mobility, and death. Through reading a large amount of literature, we have differentiated and reviewed the most common frailty scales for screening and assessment in order to help researchers as well as healthcare professionals to select the most appropriate frailty scales for its identification and assessment. Based on our summaries and generalizations, we roughly design the process of screening and assessment for the frailty in different types of older people, which is shown in Figure 1. The high-risk groups and older people without frailty symptoms are screened out through a rapid screening scale, and the high-risk groups should go to the hospital for treatment. Older people without frailty symptoms should adopt self-screening and regular community screening once every 6 months to prevent the emergence of frailty. For older hospitalized patients, high-risk groups are screened out through the doctor’s simple judgment (whether it needs to be evaluated directly), and then a detailed and comprehensive evaluation can make them quickly benefit from the follow-up personalized intervention treatment.

The development of frailty can be slowed or even reversed by early recognition, accurate assessment, and timely interventions for frailty, which will reduce the strain of frailty on healthcare systems around the world and promote healthy aging of the global population. Although we have provided insights into potential solutions for early identification and assessment of frailty by reviewing a large body of literature, however, there are some limitations to our study. Firstly, we searched only one database and limited our search to one language, which may have led to the omission of relevant articles. Secondly, our review lacked a critical assessment of the included articles, which resulted in the variable quality of the articles we included. Finally, we only summarized scales that have been studied frequently and are relatively well-established, which is somewhat one-sided. In the future, we still need to explore objective, simple, time-saving, effective, economical, and feasible scales that can accurately identify and assess frailty in clinical practice.
